# FOXC1 as a molecular predictor of postoperative peritumoral edema resolution in meningiomas

**DOI:** 10.1007/s11060-026-05620-5

**Published:** 2026-06-11

**Authors:** Alim Emre Basaran, Max Braune, Alonso Barrantes-Freer, Wolf C. Müller, Martin Vychopen, Erdem Güresir, Johannes Wach

**Affiliations:** 1 Department of Neurosurgery, University Hospital Leipzig, University of Leipzig, Liebigstr. 20, 04103 Leipzig, Germany; 2 Comprehensive Cancer Center Central Germany, Partner Site Leipzig, 04103 Leipzig, Germany; 3 Paul-Flechsig-Institute of Neuropathology, University Hospital Leipzig, 04103 Leipzig, Germany

**Keywords:** FOXC1, Meningioma, Peritumoral brain edema, Immunhistochemistry, Biomarker

## Abstract

**Background:**

Peritumoral brain edema (PTBE) is an important prognostic factor in meningiomas and may persist after tumor resection. While clinical and radiological predictors of postoperative PTBE resolution are known, the impact of molecular markers remained unclear. The aim of this study was to investigate the association of Forkhead box C1 (FOXC1) expression, as well as radiological and clinical parameters with postoperative PTBE resolution.

**Materials and methods:**

We conducted a retrospective single-center study with 75 histopathologically confirmed meningiomas. Pre- and postoperative PTBE volumes were quantified using volumetric MRI-based segmentation. Postoperative PTBE resolution was defined as the relative percentage reduction in PTBE volume compared with preoperative baseline. In addition, preoperative MRI datasets were analyzed for radiological tumor characteristics using 3D Slicer. FOXC1 expression was assessed immunohistochemically. Optimal cut-off values were determined using receiver operating characteristics (ROC) curve analysis. Univariate and multivariate analyses were performed to identify independent predictors of postoperative PTBE resolution.

**Results:**

In multivariate analysis, higher FOXC1 expression was independently associated with greater postoperative PTBE resolution (OR: 6.36, 95% CI: 2.06–19.60; *p* < 0.001). In addition, larger tumor volume was independently associated with lower PTBE resolution (OR = 0.25, 95% CI: 0.07–0.89, *p* = 0.029).

**Conclusion:**

Higher FOXC1 expression was independently associated with greater postoperative PTBE resolution. These findings require validation in larger independent cohorts before clinical implementation.

**Graphical Abstract:**

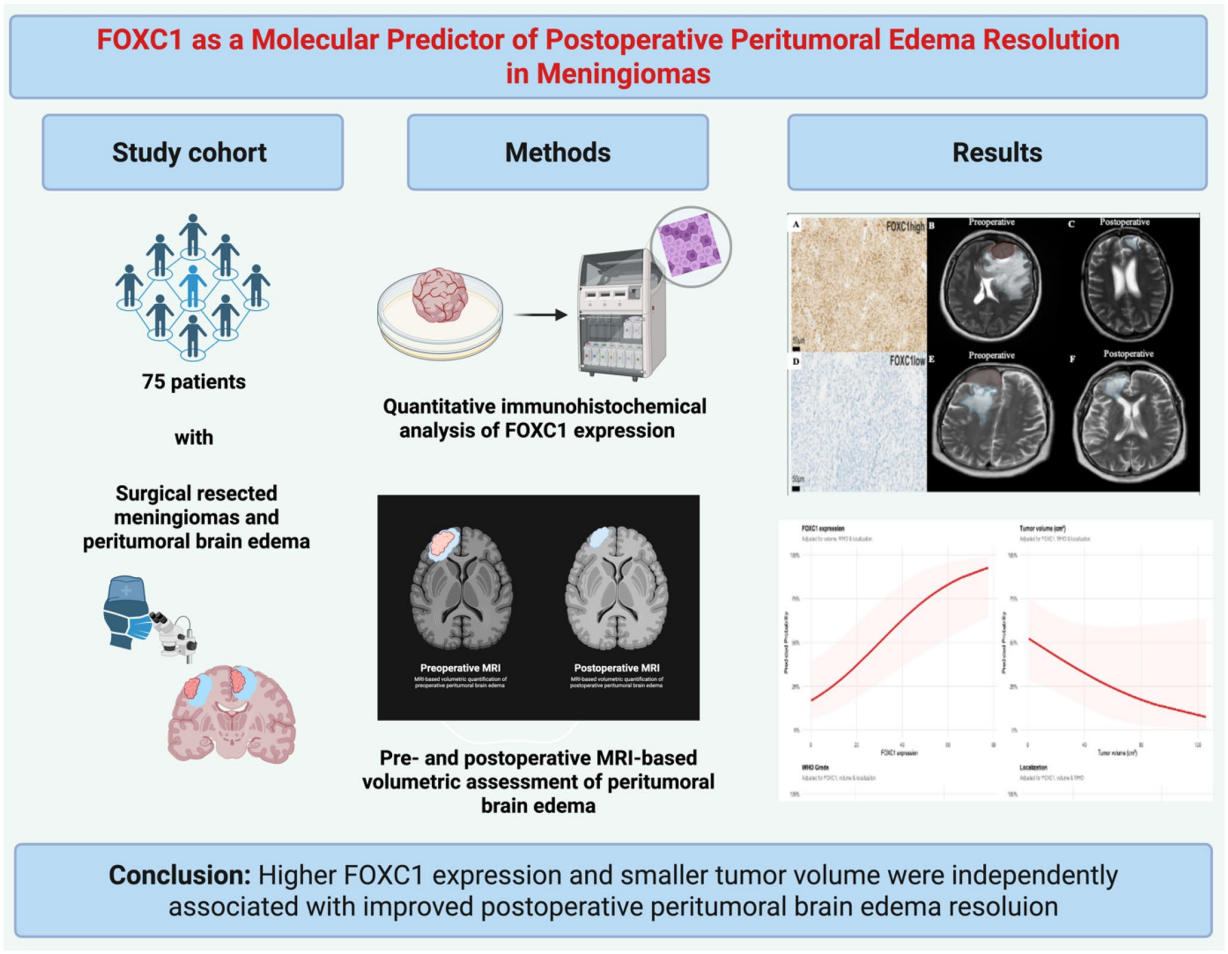

**Supplementary Information:**

The online version contains supplementary material available at 10.1007/s11060-026-05620-5.

## Introduction

Meningiomas are the most common primary intracranial tumors in adults and account for approximately 30–40% of all primary central nervous neoplasms [1–3]. Although most meningiomas are histologically benign, the clinical course can be substantially influenced by peritumoral brain edema (PTBE), which is present in half of the meningioma patients [4–6]. Extensive PTBE is associated with seizures, neurological deficits, increased perioperative morbidity, and prolonged hospitalization, and it may either persist after surgery or resolve over time [7, 8]. To date, the pathophysiology and determinants of postoperative PTBE resolution in meningioma patients remain incompletely understood.

PTBE in meningiomas is predominantly vasogenic and has been linked to blood-brain barrier (BBB) dysfunction, impaired venous drainage, and the release of vasoactive mediators [9–11]. Previous studies have identified several predictors of preoperative PTBE and postoperative edema dynamics, including tumor volume, tumor localization and morphological imaging characteristics. In addition, vascular endothelial growth factor (VEGF) has been proposed as a key mediator of edema formation, as higher VEGF expression is associated with increased vascular permeability and greater PTBE [11, 12]. 

Beyond radiological and anatomical factors recent, research has increasingly focused on molecular markers as potential predictors of PTBE dynamics. The transcription factor forkhead box C1 (FOXC1) plays a central role in vascular development and permeability, endothelial stability and regulation of the BBB [13, 14]. Experimental oncological studies have demonstrated that altered FOXC1 expression is associated with increased cell proliferation, cancer stem cell maintenance, tumor cell migration and angiogenesis [15, 16]. Recently, we demonstrated that low FOXC1 expression is associated with the presence of preoperative PTBE in meningiomas, supporting its role in tumor-associated vascular permeability and blood-brain barrier dysfunction [17]. However, whether FOXC1 expression also influences postoperative PTBE dynamics and edema resolution after tumor resection remains unknown.

In addition to its role in vascular permeability and edema formation, FOXC1 has been implicated in broader tumor-microenvironment interactions, including angiogenic signaling, endothelial remodeling and regulation of tumor-associated vascular architecture [13–15]. These mechanisms are highly relevant in the context of tumor-brain interaction and may influence not only the development but also the persistence and resolution of peritumoral brain edema. Therefore, FOXC1 may represent a molecular link between tumor biology and postoperative edema dynamics.

Therefore, the aim of the present study was to investigate the association between FOXC1 expression and postoperative PTBE resolution in meningioma patients and evaluate its potential role as a molecular biomarker linking tumor-associated vascular characteristics with postoperative edema dynamics.

## Materials and methods

This retrospective single-center observational study included patients who underwent neurosurgical intervention at the Department of Neurosurgery, University Hospital Leipzig. All patients received standardized perioperative management. Patients were included if complete data, preoperative MRI and postoperative MRI follow-up at 3 months were available, as well as sufficient tumor tissue for immunohistochemical analysis. Patients with incomplete imaging data, missing follow-up, or insufficient tissue samples were excluded. Patient selection was performed through a systematic review of institutional medical records, incorporating available clinical data and corresponding radiological imaging. Histopathological diagnoses were established in accordance with the 2016 and 2021 World Health Organization (WHO) classification of central nervous system (CNS).

### MRI evaluation of radiological tumor and PTBE characteristics

Radiological tumor characteristics including tumor surface area, roundness, flatness and tumor volume were assessed on preoperative MRI. Quantitative radiological measurements were performed using 3D Slicer software [18]. The presence and extent of PTBE were evaluated on preoperative and postoperative MRI using T2-weighted and/or FLAIR sequences, depending on availability within the respective imaging protocols. PTBE volumes were quantified using the SmartBrush tool within a semi-automatic segmentation workflow (BrainLAB^®^, Munich, Germany). Postoperative MRI was routinely performed at a standardized follow-up interval of 3 months after surgery, which served as the reference time point for assessing PTBE resolution. Furthermore, postoperative PTBE resolution was defined as the relative percentage reduction in PTBE volume between preoperative and postoperative MRI. The extent of PTBE resolution (%) was calculated as: [(preoperative PTBE volume − postoperative PTBE volume) / preoperative PTBE volume] × 100. Higher values indicate greater postoperative reduction of PTBE volume. All PTBE volumes were measured in cm³.

### Immunohistochemical detection of FOXC1

FOXC1 immunohistochemistry was carried out on 1-µm sections prepared from formalin-fixed, paraffin-embedded (FFPE) meningioma specimens. FOXC1 immunohistochemistry and digital quantification were performed according to a previously established institutional protocol. All staining steps were conducted in accordance with the routine procedures established at the Paul Flechsig Institute of Neuropathology, University Hospital Leipzig. In brief, tissue sections were incubated with a rabbit polyclonal anti-FOXC1 antibody (HPA040670; Sigma) diluted 1:50, following the manufacturer’s instructions. Automated staining was performed using the Ventana BenchMark ULTRA platform.

### Statistical analysis

All statistical analyses were performed using SPSS (Version 29; IBM, Armonk, NY, USA). For variables identified as significant in the univariate analysis, receiver operating characteristics (ROC) curve analyses were used to determine optimal cut-off values and these variables were subsequently dichotomized for further testing. Depending on data distribution and variable type, group comparisons were performed using the chi-square test or Fisher´s exact test for the univariate analysis. Variables were selected for inclusion in the multivariable regression model based on the results of univariate analyses and predefined clinical relevance to identify independent predictors of postoperative PTBE resolution. Extent of PTBE resolution was dichotomized using the median-split method [19]. In addition to dichotomized analyses, complementary Spearman correlation analyses were performed to FLAIRassess associations between continuous variables and postoperative PTBE resolution. A *p*-value of < 0.05 was considered statistically significant. ROC curves, forest plot radar plot and marginal effect plot were generated in R using the *ggplot2*,* dplyr*,* tidyr*,* tibble*,* scales*,* stringr* packages (R Foundation for statistical Computing, Vienna, Austria).

## Results

### Patient characteristics

The present study included 75 patients. Of these, 28 patients (37.3%) were male and 47 patients (62.7%) were female. The median age at surgery was 64 years (IQR: 52.0–74.0). Regarding tumor laterality, 39 tumors (52.0%) were located in the left hemisphere, 30 patients (40.0%) in the right hemisphere and 6 (8.0%) showed a bilateral hemispheric involvement. Skull-base meningiomas were present in 30 patients (40.0%), while 45 patients (60.0%) had non-skull base tumors. Perioperative seizures occurred in 15 patients (20.0%). According to the 2016 and 2021 WHO classifications, 49 patients (65.3%) had WHO grade 1, 18 (24.0%) had grade 2 and 8 (10.7%) had grade 3 meningiomas. Overall, 11 out of 75 patients (14.7%) received adjuvant radiotherapy, all of whom had WHO grade 2 and 3 tumors. Within this subgroup, 11 out of 26 patients (42.3%) underwent radiotherapy. Adjuvant radiotherapy was initiated within 6 weeks after surgery. The median MIB-1 index was 5 (IQR: 3–8) and the median FOXC1 expression was 16.75 (IQR: 3.34–37.30).

With respect to radiological tumor characteristics, the median tumor volume was 13.2 cm^3^ (3.06–30.72), the median tumor roundness was 0.40 (IQR: 0.18–0.62) and the median feret diameter was 55.62 mm (38.76–72.9) and the median postoperative residual edema volume was 7.4 cm^3^ (3.08–16.7). A detailed overview is provided in Table 1.

**Table 1:** Patient characteristics

Parameter	Value
**Sex**	
Male	28/ 75 (37.3%)
Female	47/ 75 (62.7%)
**Age at surgery (median [IQR])**	64 [52.0-74.0]
**WHO Grade**	
1	49/ 75 (65.3%)
2	18/ 75 (24.0%)
3	8/ 75 (10.7%)
**Adjuvant Radiotherapy**	
Yes	11/ 75 (14.7%)
No	64/ 75 (85.3%)
**Tumor laterality**	
Right	30/ 75 (40.0%)
Left	39/ 75 (52.0%)
Both hemispheres	6/ 75 (8.0%)
**Localization**	
Skull base	30/ 75 (40.0%)
Non skull base	45/ 75 (60.0%)
**Perioperative seizures**	
Yes	15/ 75 (20.0%)
No	60/ 75 (80.0%)
**Preoperative Dexamethasone**	
Yes	30/ 75 (40.0%)
No	45/ 75 (60.0%)
**Postoperative Dexamethasone**	
Yes	50/ 75 (66.7%)
No	25/ 75 (33.3%)
**Tumor volume in cm** ^**3**^ ** (median [IQR])**	13.2 [3.06-30.72]
**Roundness**	0.40 [0.18-0.62]
**FOXC1 expression (median [IQR])**	16.75 [3.34-37.30]
**Preoperative PTBE volume in cm** ^**3**^ ** (median [IQR])**	34.8 [20.10-65.70]
**Postoperative PTBE volume in cm** ^**3**^ ** (median [IQR])**	7.4 [3.08-16.7]
**Extent of PTBE resolution (%) (median [IQR])**	76.9 [39.5-89.7]

### Univariate analysis of PTBE resolution

#### Definition and distribution of postoperative PTBE resolution

Postoperative PTBE resolution was quantified as the relative reduction in PTBE volume based on pre- and postoperative volumetric measurements, as defined in the Materials and Methods section. The median extent of PTBE resolution in the overall cohort was 76.9% ([IQR]: 39.5–89.7). Based on this median value, patients were dichotomized into a low PTBE resolution group (< 76.9%) and a high PTBE resolution group (≥ 76.9%), which served as the outcome variable for subsequent univariate analyses.

### Evaluation of cut-off values for prediction of postoperative PTBE resolution

To determine optimal cut-off values for the prediction of postoperative PTBE resolution, receiver operating characteristic (ROC) analyses were performed for age, tumor volume, roundness, and the MIB-1 index. Optimal cut-off values were identified using the Youden index, maximizing the sum of sensitivity and specificity. After determination of the respective cut-off values, all variables were dichotomized and subsequently used in the univariate analyses. ROC analysis for age yielded an area under the curve (AUC) of 0.534 (95% CI: 0.402–0.666; *p* = 0.615). The optimal cut-off value was 62.5 years (< 62.5/ ≥ 62.5), corresponding to a sensitivity of 63.2% and a specificity of 51.4%.

**Fig. 1:**
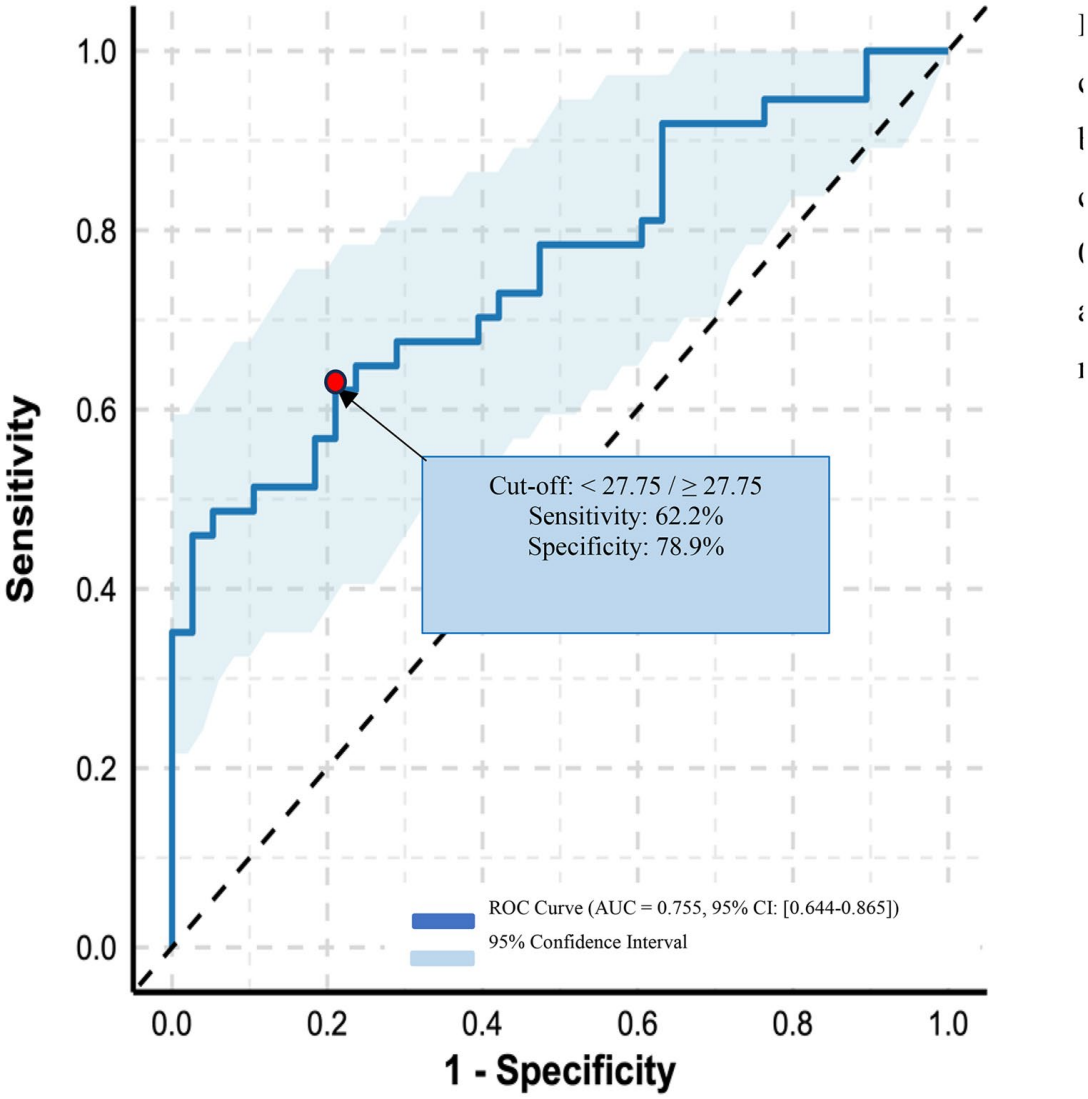
Receiver operating characteristic (ROC) curve for FOXC1 predicting postoperative peritumoral brain edema (PTBE) resolution. The area under the curve (AUC) was 0.755 (95% CI: 0.644–0.865; *p* < 0.001). At the optimal cut-off, sensitivity was 62.2% and specificity was 78.9%. The dashed line represents no discrimination

**Fig. 2:**
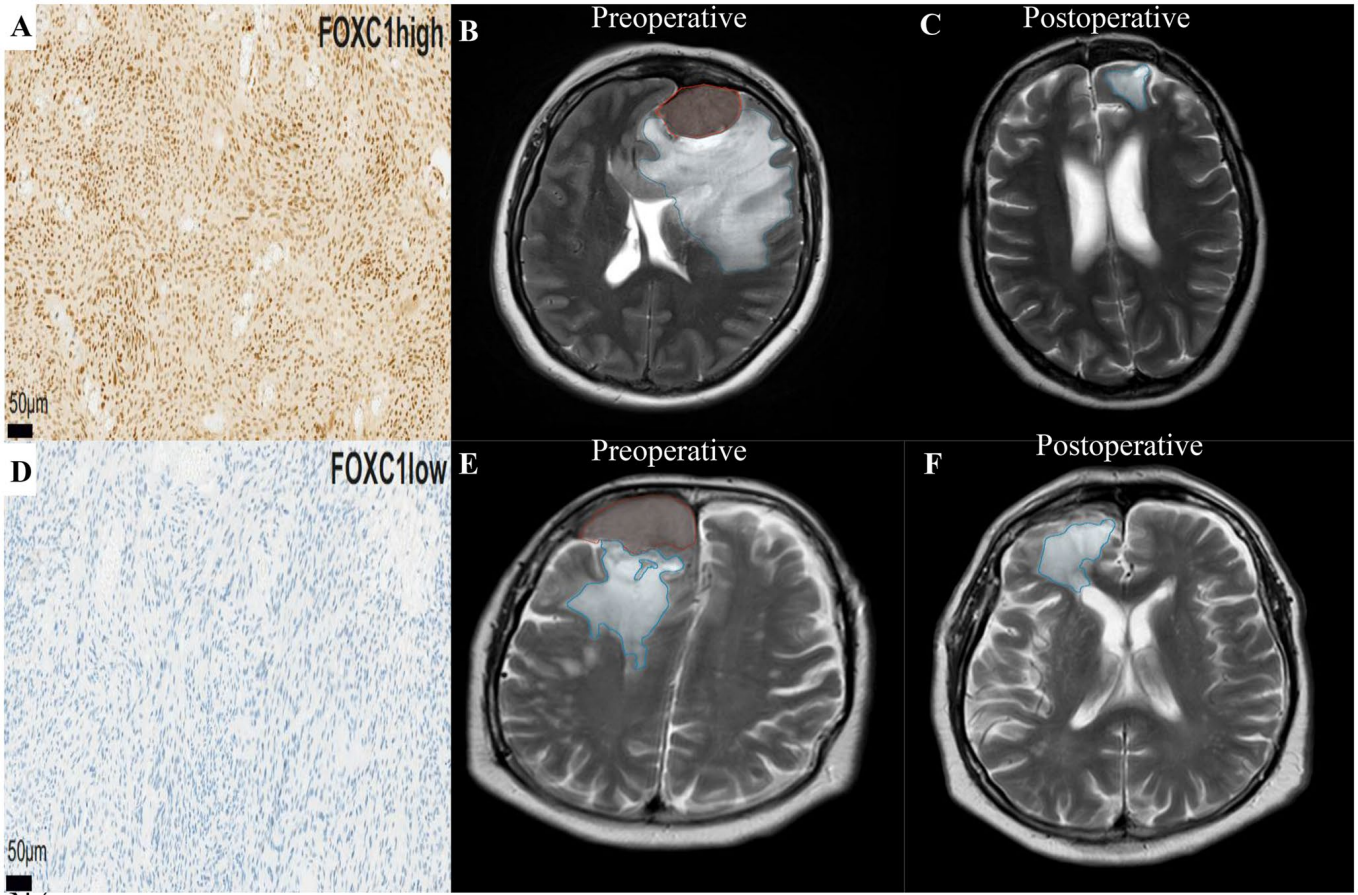
Representative examples of FOXC1 expression and postoperative PTBE resolution in meningioma patients. (**A**,** D**) Immunohistochemical staining showing high (**A**) and low (**D**) FOXC1 expression (scale bar: 50 μm). (**B**,** E**) Preoperative axial T2-weighted MRI demonstrating meningiomas with associated PTBE outlined in blue. (**C**,** F**) Postoperative axial T2-weighted MRI obtained at 3 months illustrating marked PTBE reduction in the high FOXC1 case (**C**) and limited PTBE resolution in the low FOXC1 case (**F**)

**Fig. 3:**
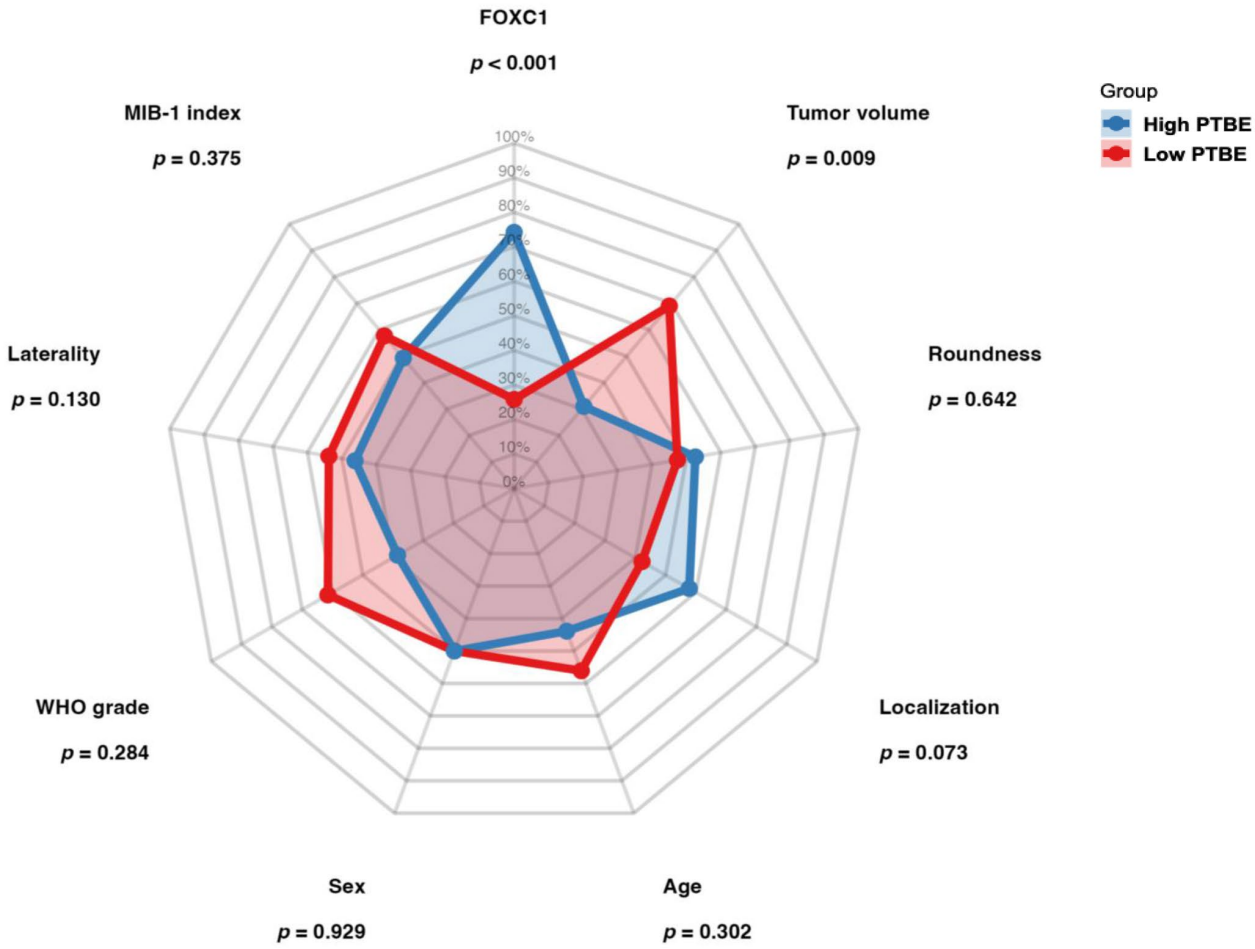
Univariate analysis of predictors associated with postoperative PTBE resolution. Radar plot comparing clinical, radiological, and molecular variables between patients with low (< 76.9%) and high (≥ 76.9%) PTBE resolution. Values represent the proportion of patients with the respective feature in each group. FOXC1 expression and tumor volume were significantly associated with PTBE resolution, whereas other variables were not

For tumor volume, the optimal cut-off was 24.55 cm³ (< 24.55/ ≥ 24.55) with an AUC of 0.615 (95% CI: 0.484–0.746; *p* = 0.089), yielding a sensitivity of 54.1% and a specificity of 75.7%. Roundness demonstrated an optimal cut-off value of 0.49 (< 0.49/ ≥ 0.49) with an AUC of 0.524 (95% CI: 0.386–0.661; *p* = 0.070) with a sensitivity of 45.9% and specificity of 78.4%. For the MIB-1 index, the optimal cut-off was 5.5 (< 5.5 / ≥ 5.5), resulting in an AUC of 0.549 (95% CI: 0.417–0.680; *p* = 0.468) with a sensitivity of 39.5% and specificity of 73.0%. The ROC curves and corresponding cut-off values, including sensitivity and specificity, are presented in Supplementary Tables 1 and Figure 1–4.

### Association between immunohistochemical FOXC1 expression and postoperative PTBE resolution

In the present study, we investigated the association between immunohistochemical FOXC1 expression in tumor tissue and postoperative radiological PTBE resolution. An optimal cut-off for FOXC1 expression was determined using ROC analysis and the variable was subsequently dichotomized. ROC analysis yielded a cut-off value of 27.75 (< 27.75/ ≥ 27.75) with an AUC of 0.755 (95% CI: 0.644–0.865; *p* < 0.001), corresponding to a sensitivity of 62.2% and a specificity of 78.9%. Figure 1 illustrates the ROC curve for FOXC1 expression in predicting postoperative PTBE resolution. Figure 2 presents representative preoperative and postoperative MRI images (T2-weighted sequences) with BrainLAB-based volumetric assessment of PTBE.

### Relationship between radiological, clinical parameters and FOXC1 and postoperative PTBE resolution

Variables included in the univariate analysis, including patient age at surgery, sex, WHO grade, tumor localization (skull base vs. non skull base), laterality, MIB-1 index, radiological parameters (roundness and tumor volume), and FOXC1 expression were selected based on their clinical relevance and biological plausibility as potential determinants of postoperative PTBE resolution. Postoperative PTBE resolution, defined as the relative reduction in PTBE volume, served as the primary outcome variable. As no standardized cut-off values exist to define clinically meaningful PTBE resolution, patients were dichotomized using the cohort median of PTBE resolution (76.9%). Accordingly, patients were classified into a low PTBE resolution group (< 76.9%, *n* = 38) and a high PTBE resolution group (≥ 76.9%, *n* = 37). For the chi-square analyses, continuous predictor variables including age at surgery, MIB-1, tumor volume, roundness, and FOXC1 expression were additionally dichotomized using optimal cut-off values derived from ROC curve analyses. These ROC analyses-based cut-offs were subsequently applied to generate binary variables for the chi-square tests.

Univariate chi-square analysis demonstrated that high FOXC1 expression (≥ 27.75) was significantly associated with high postoperative PTBE resolution, being present in 62.2% (23/37) of patients with high PTBE resolution compared with only 21.1% (8/38) in the low PTBE resolution group (*p* < 0.001). Similarly, smaller tumor volume (< 24.55 cm³) was significantly associated with high postoperative PTBE resolution, as 75.7% (28/37) of patients in the high-resolution group had smaller tumors compared with 45.9% (17/37) in the low-resolution group (*p* = 0.009). Tumor localization showed a trend toward an association with postoperative PTBE resolution. Non–skull base meningiomas were more frequently observed in the high PTBE resolution group in 70.3% (26/37) of patients compared with the low PTBE resolution group in 50% (19/38) of patients, although this association did not reach statistical significance (*p* = 0.073).

No statistically significant associations were identified for patient age at surgery (< 62.5 vs. ≥ 62.5 years; *p* = 0.302), sex (*p* = 0.929), WHO grade (*p* = 0.170), tumor laterality (*p* = 0.130), roundness (< 0.49 vs. ≥ 0.49; *p* = 0.642), or MIB-1 index (< 5.5 vs. ≥ 5.5; *p* = 0.375). Importantly, neither preoperative nor postoperative corticosteroid administration was associated with PTBE resolution, suggesting that the observed effects of FOXC1 expression are unlikely to be confounded by steroid treatment. Table 2 provides a summary of the univariate comparisons between patients with low versus high postoperative PTBE resolution and Fig. 3 illustrates these group differences graphically using a radar plot.

**Table 2:** Univariate analysis of patient characteristics, radiological parameters and FOXC1

Characteristics	Low postoperative PTBE resolution (< 76.9, *n* = 38)	High postoperative PTBE resolution (≥ 76.9, *n* = 37)	*p*-value
**Age at surgery (median [IQR])**	68.5 [51.25–73.75]	62.0 [52.0–75.0]	0.302
**WHO grade**			0.284
1	22/ 38 (57.9%)	27/ 37 (73.0%)	
2	10/ 38 (26.3%)	8/ 37 (21.6%)	
3	6/ 38 (15.8%)	2 / 37 (5.4%)	
**Sex**			0.929
Male	14/ 38 (36.8%)	14/ 37 (37.8%)	
Female	24/ 38 (63.2%)	23/ 37 (62.2%)	
**Localization**			*0.073*
Non skull base	19/ 38 (50.0%)	26/ 37 (70.3%)	
Skull base	19/ 38 (50.0%)	11/ 37 (29.7%)	
**Laterality**			0.130
Right	12/ 38 (31.6%)	18/ 37 (48.6%)	
Left	21/ 38 (55.3%)	18/ 37 (48.6%)	
Both hemispheres	5/ 38 (13.2%)	1/ 37 (2.7%)	
**Preoperative Dexamethasone**			0.300
Yes	13/ 38 (34.2%)	17/ 37 (45.9%)	
No	25/ 38 (65.8%)	20/ 37 (54.1%)	
Postoperative Dexamethasone			0.744
Yes	26/ 38 (68.4%)	24/ 37 (64.9%)	
No	12/ 38 (31.6%)	13/ 37 (35.1%)	
**MIB-1 index median [IQR])**	5 [2.5-9.0]	4.50 [2.5–6.5]	0.375
**Roundness median [IQR])**	0.39 [0.24–0.49]	0.41 [0.15–0.71]	*0.642*
**Tumor volume in cm** ^**3**^ ** median [IQR])**	25.6 [3.65–47.95]	9.38 [2.53–25.04]	*0.009*
**FOXC1**	8.03 [0.63–22.88]	35.45 [8.17–51.5]	*< 0.001*

### Multivariate analysis

Based on the results of the univariate analyses and predefined clinical relevance, variables were selected for inclusion in the multivariate binary logistic regression model to identify independent predictors of postoperative PTBE resolution. Accordingly, FOXC1 expression and tumor volume were included based on significant univariate associations, while WHO grade and tumor localization were additionally included due to their established clinical relevance [20, 21]. 

In the multivariate analysis, high FOXC1 expression (≥ 27.75) remained independently associated with higher odds of greater PTBE resolution (Odds Ratio (OR) = 6.36, 95% CI: 2.06–19.60; *p* < 0.001). In contrast, large tumor volume (≥ 24.55 cm^3^) was independently associated with lower odds of high postoperative PTBE resolution (OR = 0.25, 95% CI: 0.07–0.87; *p* = 0.029), indicating that patients with smaller tumors achieve high PTBE resolution. WHO grade (*p* = 0.761) and tumor localization (non-skull base vs. skull base; *p* = 0.169) were not statistically significant after adjustment, indicating that their univariate associations were not independent when FOXC1 expression and tumor volume were considered simultaneously. Figure 4A summarizes the results of the multivariate logistic regression model and is presented as a forest plot, while Fig. 4B illustrates the marginal effects of the independent predictors on postoperative PTBE resolution, visualizing the adjusted impact of each variable across its observed range.

### Correlation analysis

As an additional analysis, the association between FOXC1 expression and postoperative PTBE resolution was assessed using Spearman correlation. This demonstrated a significant positive relationship between FOXC1 expression and PTBE resolution (*r* = 0.423, *p* < 0.001), while tumor volume showed a significant negative correlation (*r* = -0.300, *p* = 0.009). No significant associations were observed for age (*p* = 0.294), roundness (*p* = 0.352) or MIB-1 index (*p* = 0.086). These findings are consistent with the results of the dichotomized analyses.

**Fig. 4A:**
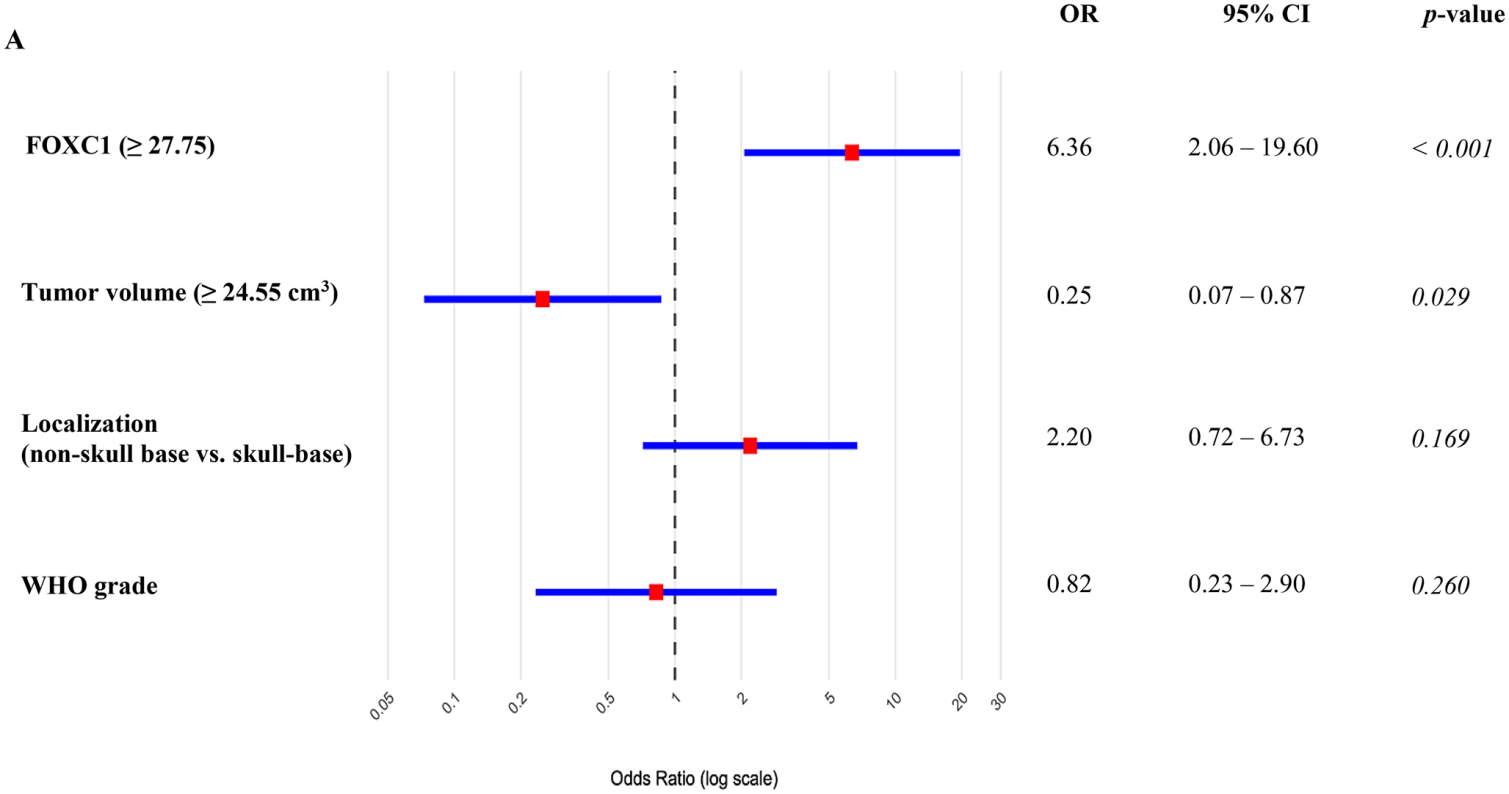
Multivariable logistic regression analysis of predictors for postoperative peritumoral brain edema (PTBE) resolution. Forest plot displaying odds ratios (ORs) with 95% confidence intervals for variables included in the model. High FOXC1 expression and smaller tumor volume were independently associated with greater PTBE resolution, whereas WHO grade and tumor localization were not significant

**Fig. 4B:**
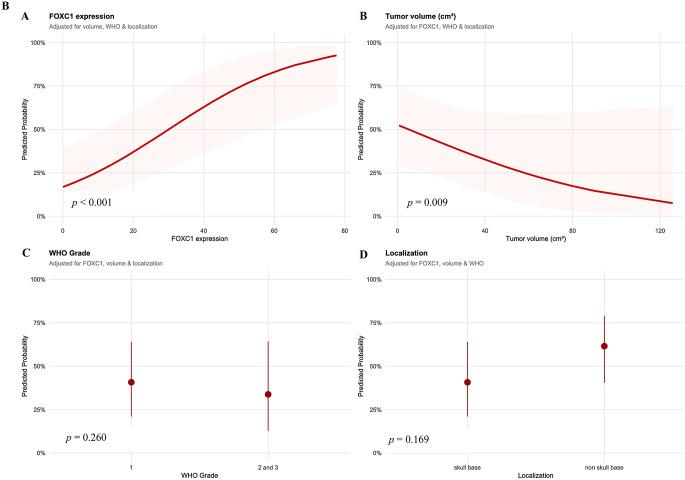
Marginal effects plots illustrating adjusted probabilities of high postoperative PTBE resolution. Marginal effects on the probability of high postoperative peritumoral brain edema (PTBE) resolution. Adjusted predicted probabilities are shown for FOXC1 expression (**A**), tumor volume (**B**), WHO grade (**C**), and tumor localization (**D**) based on the multivariable model. Shaded areas represent 95% confidence intervals

## Discussion

The aim of the present study was to investigate the association between FOXC1 expression as well as radiological and clinical parameters and postoperative PTBE resolution in histopathologically confirmed meningiomas. In contrast to our previous work, which focused on the association between immunohistochemical FOXC1 expression and preoperative PTBE occurrence, the present study extends these findings by demonstrating that FOXC1 expression is also independently associated with postoperative PTBE resolution. We found that low FOXC1 expression and larger tumor volume were significantly associated with a lower postoperative PTBE resolution. These findings should be interpreted in the context of imaging based PTBE assessment, which reflects radiological signal alterations rather than a direct measure of purely reversible vasogenic edema. Importantly, postoperative PTBE resolution represents a radiological surrogate marker rather than a direct clinical outcome. Although PTBE has been associated with neurological deficits, seizure risk and perioperative morbidity, the present study did not directly assess these clinical endpoints. Therefore, the clinical implications of the observed associations remain to be established [7, 22]. 

At first glance, the association between low FOXC1 expression and increased preoperative PTBE, together with the finding that high FOXC1 expression is associated with greater postoperative PTBE resolution, may appear contradictory. However, these findings likely reflect different aspects of tumor-associated vascular integrity. Reduced FOXC1 expression may contribute to increased vascular permeability and blood–brain barrier dysfunction, resulting in more extensive preoperative edema, as supported by experimental studies demonstrating a key role of FOXC1 in vascular development, endothelial stability, and maintenance of blood–brain barrier integrity [13, 14]. In contrast, higher FOXC1 expression may reflect more preserved vascular integrity and less structural damage, allowing for more effective normalization of the surrounding brain tissue after tumor resection. This supports the concept that FOXC1 may influence both the development and the postoperative resolution of PTBE.

To our knowledge, this is the first study to specifically evaluate FOXC1 expression as a predictor of postoperative PTBE resolution in meningioma patients. Although FOXC1 has not previously been investigated in the context of postoperative PTBE resolution in meningiomas, several studies support a biologically plausible link between FOXC1 and edema dynamics. FOXC1 is a transcription factor that plays a central role in vascular development, endothelial function and maintenance of vascular integrity. Experimental studies have demonstrated that dysregulation of FOXC1 leads to alterations in vascular architecture, increased vascular permeability and functional disruption of the basement membrane leading to endothelial barrier dysfunction [13, 14]. Given that the development and persistence of PTBE are primarily driven by microvascular permeability and BBB dysfunction, it is biologically plausible that FOXC1-dependent tumor-associated vascular characteristics influence postoperative edema resolution [9–11]. In line with this hypothesis, our results demonstrate that low FOXC1 expression was associated with reduced postoperative PTBE resolution. These findings suggest that molecular tumor characteristics, particularly FOXC1 expression could contribute to individual postoperative PTBE dynamics beyond established clinical and radiological parameters [23, 24]. FOXC1 expression should therefore not be interpreted as a direct mediator of postoperative edema resolution, but rather as a surrogate marker of tumor-associated vascular integrity and blood-brain barrier function. In this context, FOXC1 may reflect the preoperative vascular microenvironment, which could influence the extent and reversibility of PTBE following tumor resection.

In addition to molecular factors, tumor volume emerged as an independent predictor of postoperative PTBE resolution. In the present study, larger tumor volume was significantly associated with lower postoperative PTBE resolution. This finding is consistent with previous studies reporting an association between increased tumor burden and more pronounced or persistent PTBE. Pathophysiologically, larger tumors are thought to induce greater mechanical compression of surrounding brain tissue, impair venous drainage and exacerbate BBB dysfunction, thereby promoting sustained edema formation [10, 25]. Previous studies have shown that a higher edema to tumor ratio represents an unfavorable prognostic marker and is associated with poorer clinical outcomes [26]. These observations are in accordance with the concept that PTBE in meningiomas is largely driven by vascular permeability and the release of vasoactive mediators. In this context, VEGF has been identified as a key mediator of vasogenic edema formation [11]. Otsuka et al.^12^ demonstrated a correlation between edema volume and edema index with VEGF and VEGF receptor expression in meningiomas. Additional studies have shown that VEGF increases vascular permeability and promotes vasogenic brain edema [27, 28]. Taken together, these data support our findings that larger tumor volumes, often accompanied by increased angiogenic activity and higher edema burden are associated with slower or incomplete postoperative normalization of the postoperative tumor bed environment, resulting in reduced PTBE resolution. In addition to VEGF, several other molecular pathways have been implicated in the pathophysiology of PTBE in meningiomas. Matrix metalloproteinases, particularly MMP-9, are known to contribute to extracellular matrix degradation and increased vascular permeability, thereby facilitating edema formation [11, 29]. Similarly, CD44 has been associated with tumor cell adhesion, migration and interactions with the extracellular matrix, potentially influencing tumor-associated edema [30]. Furthermore, aquaporin-4, a key water channel protein expressed in astrocytic endfeet, plays a critical role in water homeostasis and has been linked to the development and persistence of vasogenic brain edema [31]. In this context, FOXC1 may be viewed as part of a broader regulatory network affecting vascular integrity, endothelial function and blood-brain permeability. While these pathways were not directly assessed in the present study, they highlight the multifactorial nature of PTBE and provide a broader biological context for the observed association with FOXC1 expression.

Our findings may have potential clinical implications for risk stratification and perioperative management of meningioma patients. Impaired postoperative PTBE resolution is clinically relevant, as persistent edema has been associated with neurological deficits, prolonged hospital stays, increased perioperative morbidity and higher mortality rates [7, 32]. In this context, the integration of molecular markers such as FOXC1 with established radiological predictors including tumor volume and localization may potentially help to identify patients at risk for delayed or incomplete PTBE resolution. Such patients may require closer postoperative monitoring, earlier or standardized follow-up imaging and individualized symptom management strategies. Importantly, several studies have demonstrated that the extent of PTBE is one of the strongest predictors of tumor-related epilepsy, both preoperatively and postoperatively. Large-scale observational studies and recent meta-analyses have shown that patients with extensive PTBE have a significantly higher risk of seizures, independent of tumor size, localization, or WHO grade [22, 33–35]. These findings underscore that PTBE is not merely a secondary imaging phenomenon but a major driver of neurological morbidity in meningioma patients.

Despite its major clinical relevance, therapeutic options to actively modulate PTBE remain remarkably limited. In current clinical practice, perioperative edema management relies almost exclusively on corticosteroids, most commonly dexamethasone [28, 36]. While steroids can effectively reduce vasogenic edema in some patients, treatment response is highly variable and difficult to predict [11, 37]. Moreover, prolonged or high-dose corticosteroid therapy is associated with substantial side effects, including hyperglycemia, psychiatric symptoms, and impaired wound healing [10]. Importantly, no established molecular or imaging-based biomarkers currently exist to identify patients at risk for persistent edema or poor steroid responsiveness [28, 37]. In this therapeutic landscape, FOXC1 may represent a biologically meaningful biomarker linking tumor-associated vascular integrity, edema formation, and postoperative edema resolution [13, 14]. These mechanisms are directly implicated in the pathophysiology of vasogenic brain edema and provide a biologically plausible explanation for our observation that low FOXC1 expression is associated with delayed postoperative PTBE resolution [28]. 

From a clinical perspective, these findings are particularly relevant for patients at increased risk of seizure activity and prolonged steroid dependency [22, 35]. Persistent postoperative edema has been associated with ongoing neurological symptoms and delayed functional recovery, often necessitating extended corticosteroid treatment [38, 39]. FOXC1-based stratification may therefore help to identify patients who require closer postoperative monitoring, more cautious steroid tapering, or intensified follow-up imaging. Furthermore, patients with low FOXC1 expression could represent a subgroup in whom alternative or adjunctive anti-edematous strategies, such as anti-angiogenic approaches, warrant further investigation [37]. Importantly, meningiomas are extra-axial tumors that do not originate from brain parenchyma and PTBE is thought to arise primarily from tumor-brain interface mechanisms rather than direct tumor invasion [10]. In this context, FOXC1 expression should not be interpreted as a direct parenchymal drive of edema. Instead, FOXC1 may reflect tumor-associated vascular characteristics, including endothelial stability, angiogenesis and vascular permeability, which can influence the tumor microenvironment and its interaction with the surrounding brain tissue. This interpretation is supported by experimental studies demonstrating a key role of FOXC1 in vascular development, endothelial integrity and maintenance of blood-brain barrier function [13, 14]. Disruption of the tumor-brain interface, including potential alterations of the pia mater, may represent a key mechanism linking extra-axial tumors to PTBE. Although such parameters were not assessed in the present study, they may provide an important avenue for future investigations combining molecular, radiological and intraoperative data.

Several limitations of the present study should be acknowledged. First, postoperative FLAIR hyperintense signal alterations may not exclusively represent residual vasogenic edema. Persistent hyperintensity around the resection cavity at 3 months may also reflect irreversible tissue alterations, including gliosis, ischemic injury, postoperative reactive changes or chronic parenchymal damage resulting from long-standing preoperative edema [10]. Therefore, postoperative PTBE resolution in this study should be interpreted as radiological resolution of PTBE-associated FLAIR signal abnormality rather than as a direct measure of purely reversible edema. Second, postoperative PTBE may also be influenced by surgical factors, including intraoperative manipulation, vascular compromise, and postoperative ischemic changes, which may manifest as abnormalities on diffusion-weighted imaging (DWI). These factors were not systematically assessed in the present study and may have affected postoperative FLAIR signal alterations and the estimation of PTBE resolution. In addition, postoperative MRI examinations were partially performed externally and imaging protocols were therefore not fully standardized, particularly regarding the use of T2-weighted versus FLAIR sequences. This may have introduced variability in the radiological assessment of PTBE. Third, although no tumor regrowth or progression was observed within the 3-month postoperative follow-up period, the potential influence of adjuvant radiotherapy on postoperative PTBE dynamics cannot be excluded, particularly as treatment was initiated within the early postoperative period. This may be especially relevant in higher-grade meningiomas, where radiotherapy is more frequently applied and may affect edema dynamics through radiation-induced changes in vascular permeability and tissue integrity [40, 41]. Fourth, the present study is a retrospective single-center study with a small sample size, which may introduce selection bias and limits the generalizability of subgroup analyses, particularly regarding WHO grade and tumor localization. In addition, the relatively high proportion of WHO grade 2 and 3 meningiomas in the present cohort may reflect selection bias related to the study inclusion criteria and the tertiary referral nature of our institution, which may further limit generalizability to an unselected meningioma population. In addition, histological heterogeneity, particularly the presence of secretory or angiomatous meningiomas, may influence PTBE formation and resolution and was not specifically analyzed in the present study, representing a potential confounding factor. Furthermore, given the limited sample size in relation to the number of candidate predictors, the risk of overfitting in the multivariable model cannot be fully excluded. Fifth, postoperative MRI follow-up was limited to three months, reflecting short-term PTBE dynamics and precluding assessment of long-term edema resolution. Sixth, the dichotomization of continuous variables, including PTBE resolution and FOXC1 expression, may have reduced statistical power and led to a loss of information. However, given the exploratory nature of this study and the lack of established clinically meaningful cut-off values, this approach was applied to enhance interpretability and to identify potential threshold for clinical risk stratification. Importantly, supplementary analysis using continuous variables confirmed the observed association between FOXC1 expression and postoperative PTBE resolution, supporting the robustness of the findings. Seventh, despite multivariate adjustment, residual confounding due to perioperative factors cannot be fully excluded. Finally, FOXC1 expression was assessed using immunohistochemistry, quantitative mRNA or protein analyses were not performed and may provide additional insight, although immunohistochemical assessment itself may be subject to interobserver and preanalytical variability.

## Conclusion

This study demonstrates that higher FOXC1 expression and smaller tumor volume were independently associated with greater postoperative PTBE resolution in meningioma patients. These findings suggest that FOXC1 may represent a potential molecular marker of postoperative edema dynamics in addition to established radiological parameters. However, the clinical relevance of these findings requires validation in larger, independent prospective cohorts before potential clinical application.

## Supplementary Information

Below is the link to the electronic supplementary material.

Supplementary Material 1

## Data Availability

No datasets were generated or analysed during the current study.
