# Supplementary material for: FOXC1 as a molecular predictor of postoperative peritumoral edema resolution in meningiomas
Source: J Neurooncol. 2026 Jun 11;178(2):59. doi: 10.1007/s11060-026-05620-5 (PMC13260224; doi:10.1007/s11060-026-05620-5)
Supplement: Supplementary file 1 — Supplementary Material 1 [file 11060_2026_5620_MOESM1_ESM.docx]

**Supplementary materials**

**Supplementary Table 1.** Optimal cut-off values of variables defined by ROC curves

| Variables | Cut-off values | AUC | 95% CI | Sensitivity | Specificity | *p*-value |
| --- | --- | --- | --- | --- | --- | --- |
| Age | < 62.5/ ≥ 62.5 | 0.534 | 0.402–0.666 | 63.2% | 51.4% | 0.615 |
| Tumor volume | < 24.55/ ≥ 24.55 | 0.615 | 0.484–0.746 | 54.1% | 75.7% | 0.089 |
| Roundness | < 0.49/ ≥ 0.49 | 0.524 | 0.386–0.661 | 45.9% | 78.4% | 0.070 |
| MIB-1 index | < 5.50/ ≥ 5.50 | 0.549 | 0.417–0.680 | 39.5% | 73.0% | 0.468 |

**Supplementary Figure 1**. Receiver operating characteristic (ROC) curve illustrating the diagnostic performance of age at surgery for predicting postoperative peritumoral brain edema (PTBE) resolution. Age was dichotomized at an optimal cut-off of 62.5 years (<62.5 vs. ≥62.5 years). The area under the curve (AUC) was 0.534 (95% CI: 0.402–0.666; *p* = 0.615), indicating no discriminative ability. At this cut-off, sensitivity was 63.2% and specificity was 51.4%. The solid blue line represents the ROC curve, the dashed diagonal line indicates no-discrimination performance, and the shaded area denotes the 95% confidence interval.


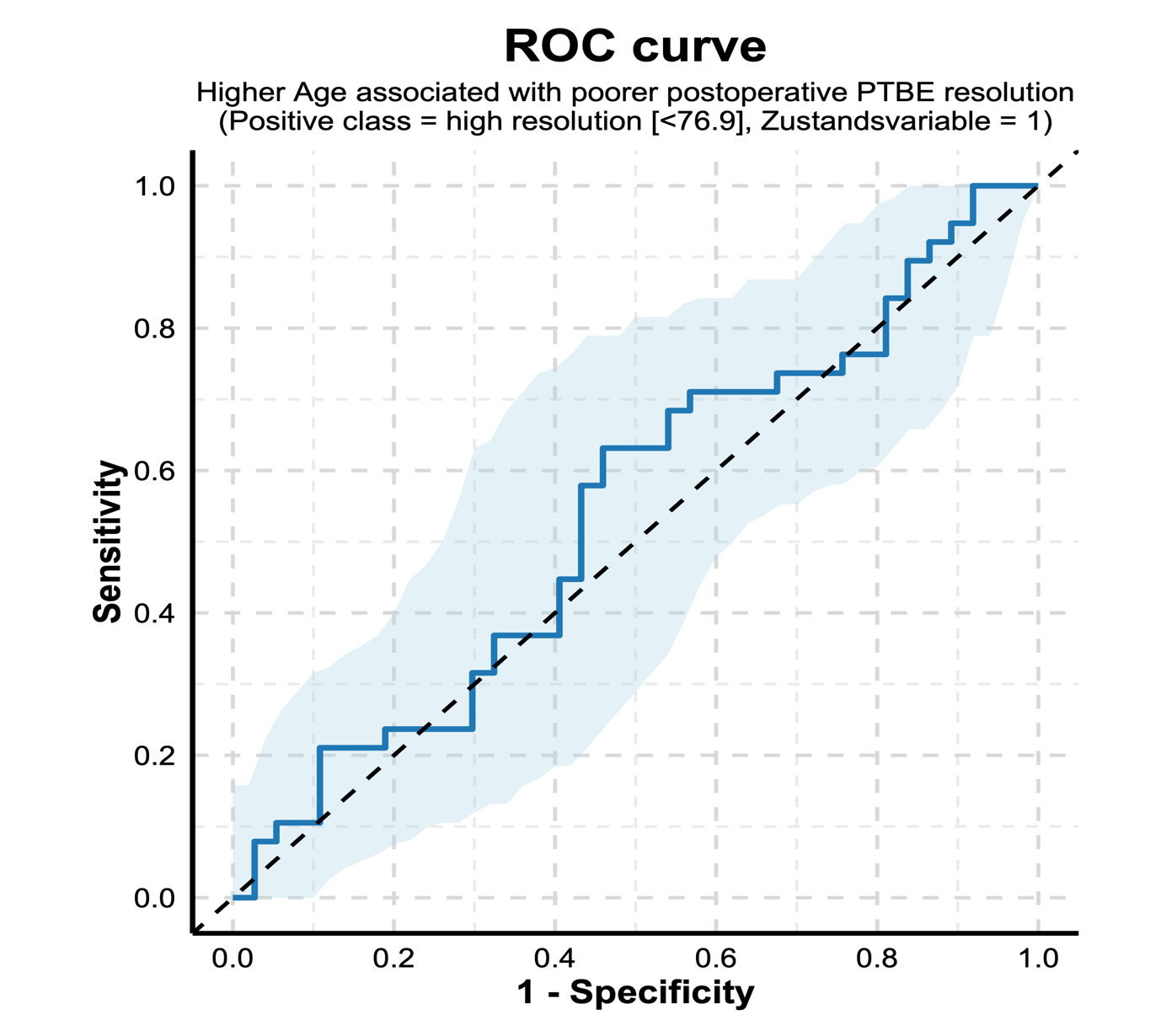


Cut-off: < 62.5/ ≥ 62.5

Sensitivity: 63.2%

Specificity: 51.4%

ROC Curve (AUC = 0.534, 95% CI: [0.402-0.666])

95% Confidence Interval

**Supplementary Figure 2**. Receiver operating characteristic (ROC) curve showing the discriminative performance of tumor volume for predicting postoperative peritumoral brain edema (PTBE) resolution. Tumor volume was dichotomized at an optimal cut-off of 24.55 cm³ (<24.55 vs. ≥24.55 cm³). The area under the curve (AUC) was 0.615 (95% CI: 0.484–0.746; *p* = 0.089), indicating moderate but non-significant discriminative ability. At this threshold, sensitivity was 54.1% and specificity was 75.7%. The solid blue line represents the ROC curve, the dashed diagonal line indicates chance-level discrimination, and the shaded area denotes the 95% confidence interval.


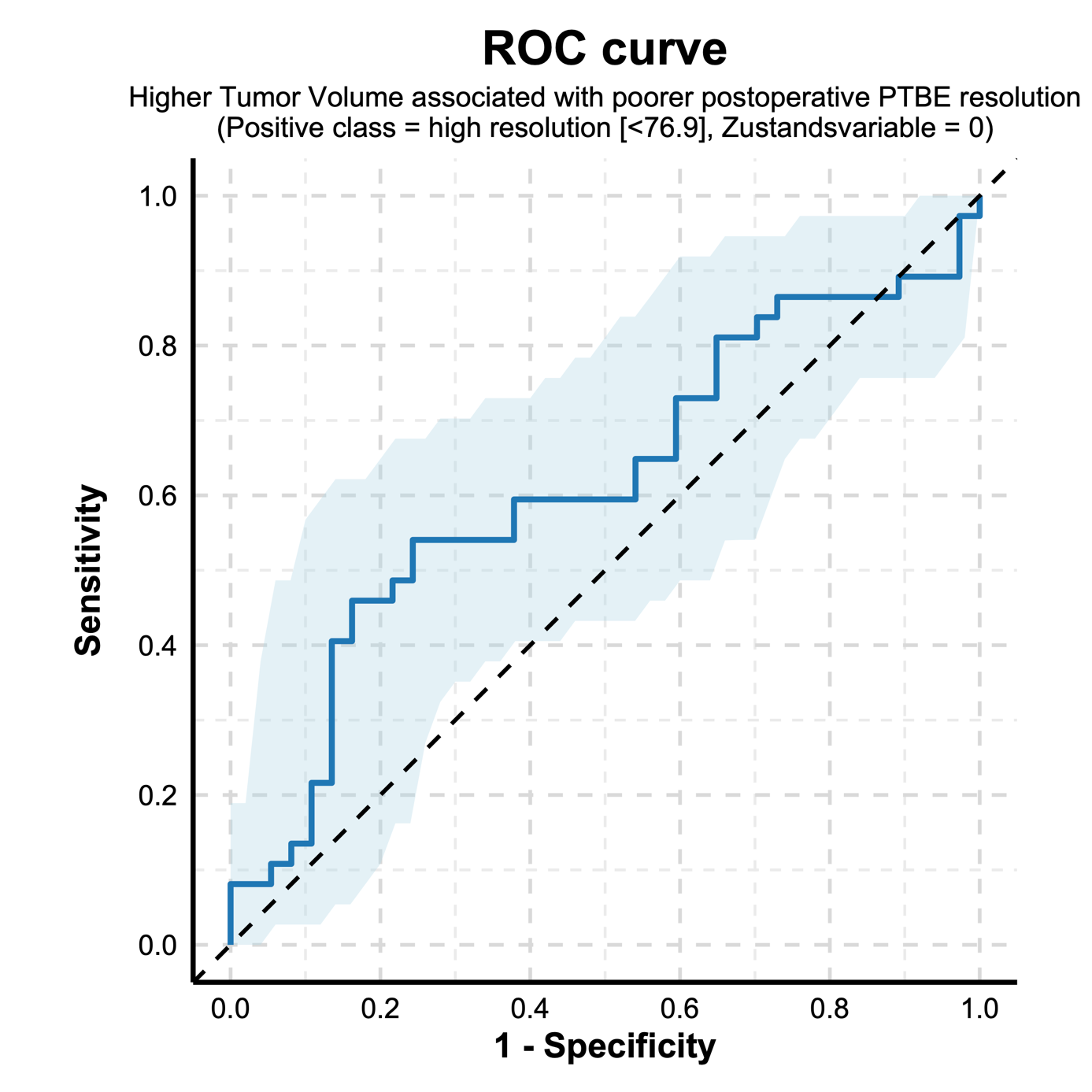


Cut-off: < 24.55 / ≥ 24.55

Sensitivity: 54.1%

Specificity: 75.7%

ROC Curve (AUC = 0.615, 95% CI: [0.484-0.746])

95% Confidence Interval

**Supplementary Figure 3.** Receiver operating characteristic (ROC) curve illustrating the predictive performance of tumor roundness for postoperative peritumoral brain edema (PTBE) resolution. Tumor roundness was dichotomized at an optimal cut-off value of 0.49 (<0.49 vs. ≥0.49). The area under the curve (AUC) was 0.524 (95% CI: 0.386–0.661; *p* = 0.07), indicating limited and non-significant discriminative ability. At this threshold, sensitivity was 45.9% and specificity was 78.4%. The solid blue line represents the ROC curve, the dashed diagonal line indicates no discrimination, and the shaded area denotes the 95% confidence interval.


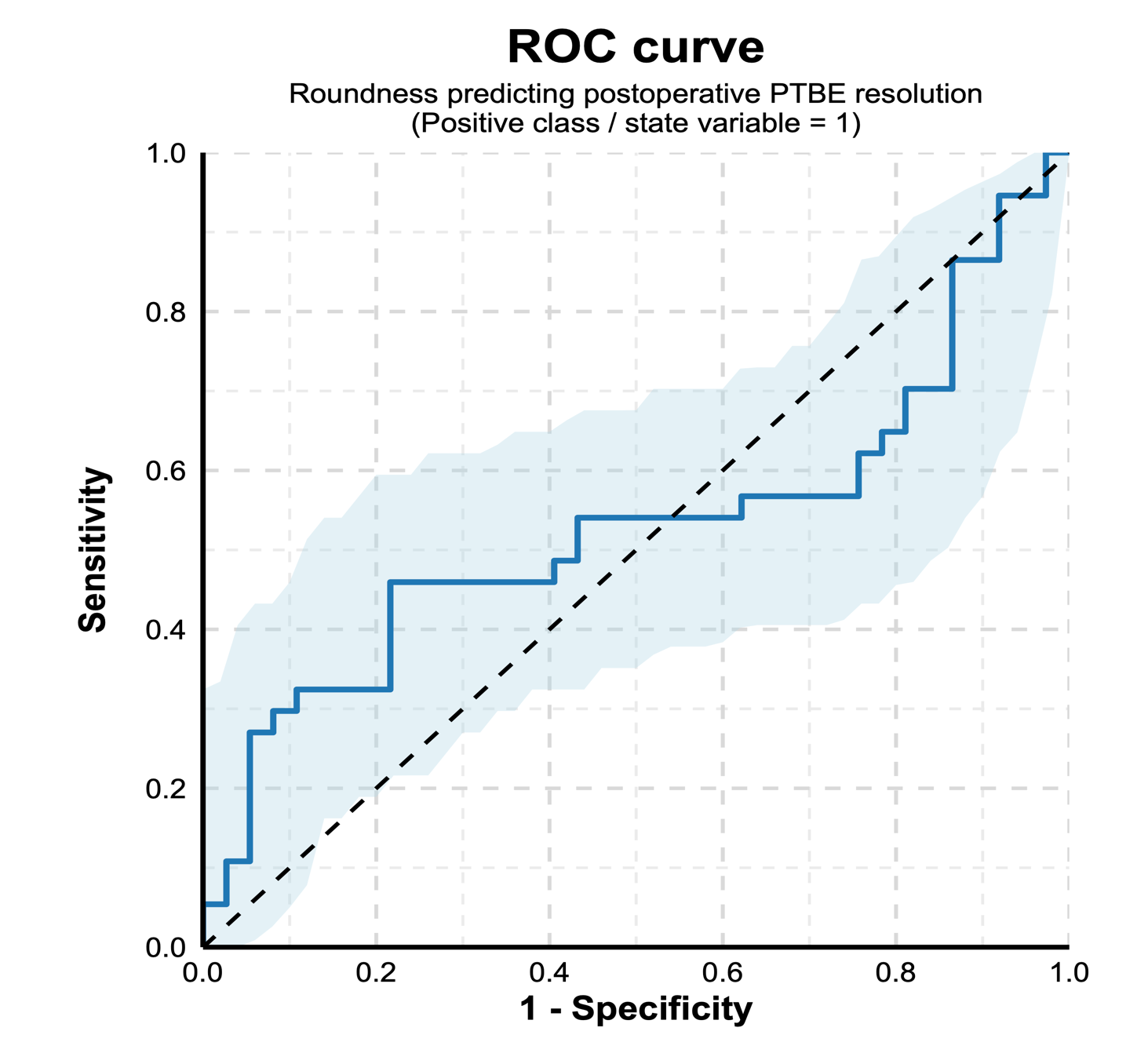


Cut-off: < 0.49 / ≥ 0.49

Sensitivity: 45.9%

Specificity: 78.4%

ROC Curve (AUC = 0.524, 95% CI: [0.386-0.661])

95% Confidence Interval

**Supplementary Figure 4**. Receiver operating characteristic (ROC) curve showing the discriminatory performance of the MIB-1 (Ki-67) index for postoperative peritumoral brain edema (PTBE) resolution. The MIB-1 index was dichotomized at an optimal cut-off value of 5.50 (<5.50 vs. ≥5.50). The area under the curve (AUC) was 0.549 (95% CI: 0.417–0.680; p = 0.468), indicating poor and non-significant discrimination. At this threshold, sensitivity was 39.5.9% and specificity was 73.0%. The solid blue line represents the ROC curve, the dashed diagonal line indicates no discriminative ability, and the shaded area denotes the 95% confidence interval.


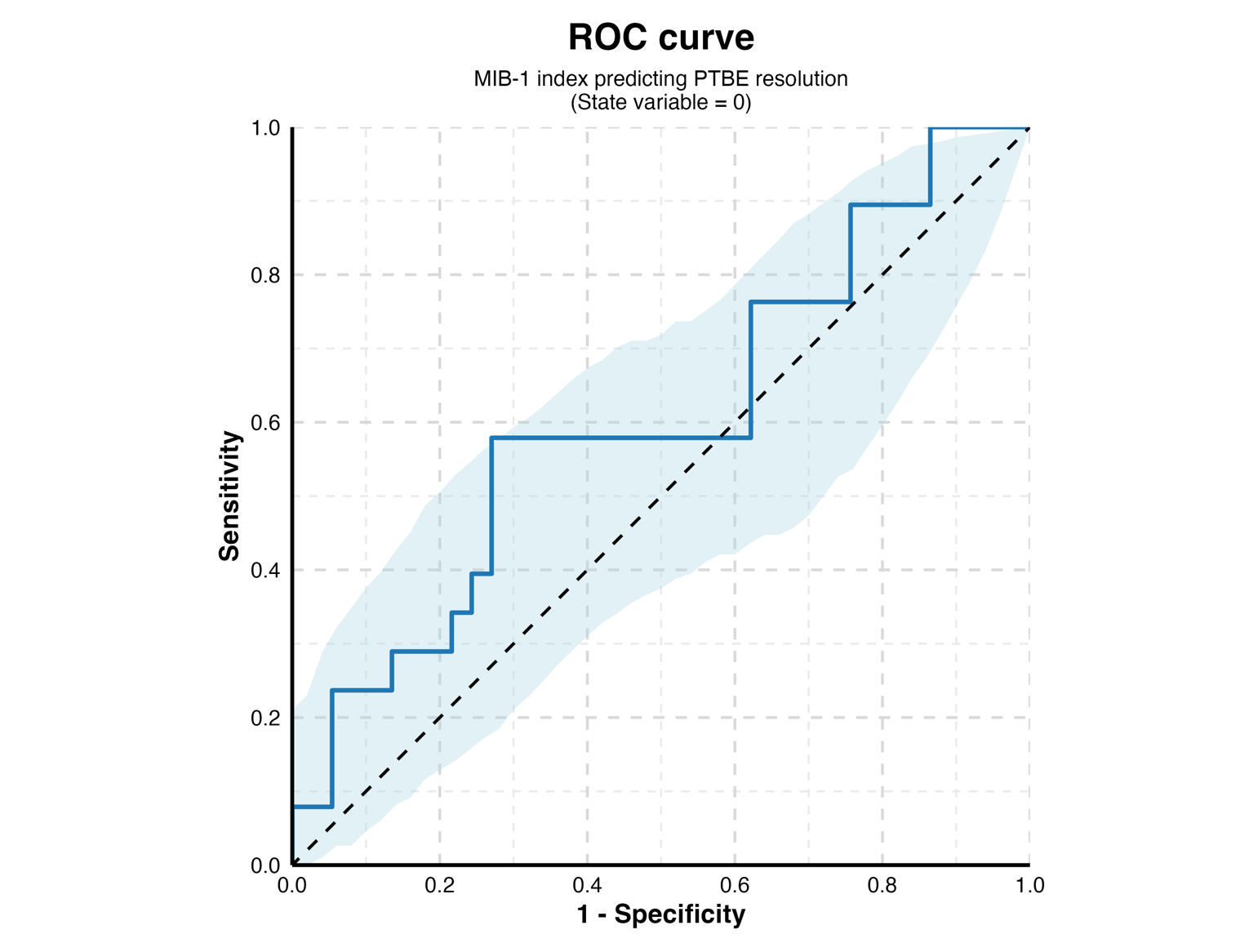


ROC Curve (AUC = 0.549, 95% CI: [0.417-0.680])

95% Confidence Interval

Cut-off: < 5.5 / ≥ 5.5

Sensitivity: 39.5%

Specificity: 73.0%
